# Participant compensation in global health research: a case study

**DOI:** 10.1093/inthealth/ihaa064

**Published:** 2020-11-09

**Authors:** Sepeedeh Saleh, Henry Sambakunsi, Deborah Nyirenda, Moses Kumwenda, Kevin Mortimer, Martha Chinouya

**Affiliations:** Malawi-Liverpool-Wellcome Trust, Queen Elizabeth Central Hospital, College of Medicine, P.O. Box 30096, Chichiri, Blantyre 3, Malawi; Liverpool School of Tropical Medicine, Pembroke Place, Liverpool L3 5QA, UK; Malawi-Liverpool-Wellcome Trust, Queen Elizabeth Central Hospital, College of Medicine, P.O. Box 30096, Chichiri, Blantyre 3, Malawi; Malawi-Liverpool-Wellcome Trust, Queen Elizabeth Central Hospital, College of Medicine, P.O. Box 30096, Chichiri, Blantyre 3, Malawi; Malawi-Liverpool-Wellcome Trust, Queen Elizabeth Central Hospital, College of Medicine, P.O. Box 30096, Chichiri, Blantyre 3, Malawi; Liverpool School of Tropical Medicine, Pembroke Place, Liverpool L3 5QA, UK; Liverpool School of Tropical Medicine, Pembroke Place, Liverpool L3 5QA, UK

**Keywords:** ethics dumping, global health, participant compensation, research ethics

## Abstract

**Background:**

Compensation for research participants can be provided for reasons including reimbursement of costs; compensation for time lost, discomfort or inconvenience; or expression of appreciation for participation. This compensation involves numerous ethical complexities, at times entailing competing risks. In the context of transnational research, often incorporating contexts of economic inequality, power differentials and post-colonialism, these issues extend into wider questions of ethical research conduct.

**Methods:**

We describe experiences of conducting a community-based study of air pollution in southern Malawi incorporating ethnographic, participatory and air quality monitoring elements. Decisions surrounding participant compensation evolved in response to changing circumstances in the field.

**Results:**

Attention to careful researcher–participant relationships and responsiveness to community perspectives allowed dynamic, contextualised decision-making around participant compensation. Despite widely cited risks, including but not restricted to undue influence of monetary compensation on participation, we learned that failure to adequately recognise and compensate participants has its own risks, notably the possibility of ‘ethics dumping’.

**Conclusions:**

We recommend active engagement with research participants and communities with integration of contextual insights throughout, including participant compensation, as for all elements of research conduct. Equitable research relationships encompass four central values: fairness, care, honesty and respect.

## Introduction

Transnational health research has grown exponentially in the last 10 years.^1^ An accompanying increase in the scrutiny of researcher–participant relationships, on both the macro and micro levels, has led to questions around who benefits from research projects: questions now at the forefront of academic debates.^2^^–^^5^ The ethical question of ‘value’ in medical research, articulated in terms of ‘a negotiation between the interests of communities, the protocols of science, the priorities of global health’,^6^ is fundamental in considering what constitutes good research conduct.

Research carried out in low- or middle-income countries by researchers from high-income country institutions implicates a population who are often comparatively disempowered and economically vulnerable. This dynamic enables the practice of ethics dumping, described as ‘the export of unethical research practices from a high-income to a resource-poor setting’.^7^ Ethics dumping may take the form of export of research for the purposes of eluding strict ethical regulations or may be more subtle. Such cases include researchers applying lower standards of ethical scrutiny in the belief that their work is beneficial to vulnerable populations, particularly in low-income settings, or a lack of attention to sociocultural values in their research settings.^8^

Individuals considering participating in research balance the risks of harm with the potential benefits. Such benefits may include direct benefits from study interventions, indirect/collateral benefits, e.g. healthcare or monetary payments, or aspirational benefits arising from the products of the study, e.g. new vaccines.^9^ Collateral benefits stand out particularly for people living with economic insecurity. We can distinguish between three types of payments: reimbursement for expenses incurred or loss of wages, payments incentivising participation and payments to demonstrate appreciation for participants’ involvement.

In settings of widespread economic vulnerability, decisions around provision of financial payments or goods and/or services can be complex. Ethics dumping here could represent researchers failing to fully value participants’ research contributions and thus providing inadequate compensation or by allowing monetary payments or other influences to increase participation among communities who would otherwise be opposed to involvement—so-called undue influence.^10^^–^^12^ Additional concerns around participant compensation, again rooted in wider contextual inequities, include risks of comparatively large payments disrupting household or local dynamics or adversely affecting local researchers through systemic inflation.^13^^,^^14^ These concerns reflect a contested field with decisions of what constitutes best practice unclear.

A final point, relevant to the debate on participant and community compensation and wider aspects of research practice, is that poorly conducted transnational research fails to respect the sociocultural values of ‘researched’ communities, leaving people open to exploitation and mistreatment. Such ethics dumping practices risk furthering existing inequalities and reinforcing historically and politically shaped extractive relationships. The imperative in transnational research to consider the benefits for potential participants and their communities is therefore paramount.

This article presents the experiences of a research collaboration between the UK and Malawi conducting a mixed methods study of air pollution in Malawi, with reference to participant compensation and related ethical issues. We draw on the existing literature to situate our experiences and thereby contribute to the wider ethical debates on transnational health research.

## Methods

### Study outline

Entitled Pamodzi, meaning ‘together’ in Chichewa (the main Malawian language), this ethnographic project applied participant observation, air quality monitoring and participatory approaches to the issue of air pollution in one village on the outskirts of Blantyre.

The research team comprised a female British PhD candidate, male Malawian research assistant and female Malawian fieldworker, herself a resident in the study village. The study was based at an international research institution in Malawi, which hosts many researchers and projects originating outside the African continent.

Pamodzi aimed at understanding the role of smoke within village life, how ‘air pollution’ is prioritised within daily concerns (if at all) and describing differential smoke exposures across the community. Workshops then brought together residents to develop context-appropriate ‘clean air’ solutions. The resulting intervention—a locally produced clay cookstove for all households and recommendations for cooking to take place in well-ventilated spaces—will be piloted across the village in the next phase. The project is deeply rooted in the local village community, potentially involving all consenting residents.

### Study setting

Malawi, in southern Africa, has a population of approximately 19 million.^15^ Most of the population are rural, with widespread poverty. The stated minimum wage is 1,346 Malawian Kwacha (MK) per day (approximately US$1.30), although only around 1 in 10 Malawians are formally employed. Most are subsistence farmers with additional ad hoc piecework or self-employment providing extra income.^16^^,^^17^ In recent years, poverty and subsequent food insecurity have been exacerbated by floods and droughts that threaten to worsen with the advancing climate crisis.^18^^–^^20^ Thus economic limitation and precarity is important in individuals’ lifestyles (e.g. with access to electricity being very limited) and in shaping researcher–participant power differentials, specifically in terms of participant compensation.

During the colonial period and beyond, biomedical research, lacking the current ethical regulations and safeguards, employed various exploitative and dishonest practices. Accounts of information concealment and the use of force, often through local chiefs, to compel individuals into participation are widespread.^21^^,^^22^ In Malawi, beliefs about ‘bloodsuckers’—rumours involving the stealing of blood through witchcraft, or its removal using modern technology, with subsequent witchcraft-related uses—are ever-present, accompanying many medical and research projects (although not, to our knowledge, Pamodzi).^23^ While at times seemingly simple responses to uncertainty, e.g. around food insecurity, the underlying belief systems are likely, at least in part, to stem from colonial power dynamics and transnational research practices.^22^^–^^24^ This is particularly relevant where bloodsucker rumours are aimed at overseas researchers and non-governmental organization staff. Analyses of this issue point to histories of extractive imperial practices, with blood often felt to represent the ‘life force’, and accumulation of unexplained wealth: reification of the inequality inherent in these relationships.^24^

Malawi gained independence in 1964, but legacies of colonial practices—including the use of power to impose medical and research interventions on colonised populations without their fully informed consent^25^^,^^26^—are ever-present, and relevant when planning and practising transnational collaborative research.

### Study methods

The Pamodzi village-based study used local introductions, starting with the chief and group village head (overall chief of a wider area), then the community health volunteer and other key village members, including religious leaders. Discussions with residents followed over a 1-month period, prompting conversations around the project concept, acceptability and implementation. Dialogue with the chief and community elders in this period led to a form of community approval that prefaced the ensuing consent processes.^27^ Recruitment discussions involving iterative, personalised consultations also took place during and beyond this phase.

Individual and household consent used information leaflets and consent forms alongside verbal explanations. An extended process over at least two time points arranged individually with participants allowed for careful consideration by all parties and true freedom to withdraw. Walk-around consultations in the village at various points during the study ensured community engagement and ongoing consent throughout.

Study methods included participant observation in and around individual households and extended throughout the village and personalised air quality monitoring. Purposeful sampling was used for recruitment: selected households varied by size, gender of household head and other features and individuals involved in air quality monitoring were recruited to reflect variations in lifestyles and exposures.^28^ The subsequent participatory methods lie outside the scope of this article. A summary of study components and participants is provided in Table 1.

**Table 1.** Components of the study and participants included in each

Study component	Participants
General village-based participant observation	Whole village potentially involved, >3000 people
Focused household participant observation	Members of six households
Additional individual air quality monitoring	20 adults
Participatory workshops (6 in total)	16 adults per workshop

**Figure 1.**
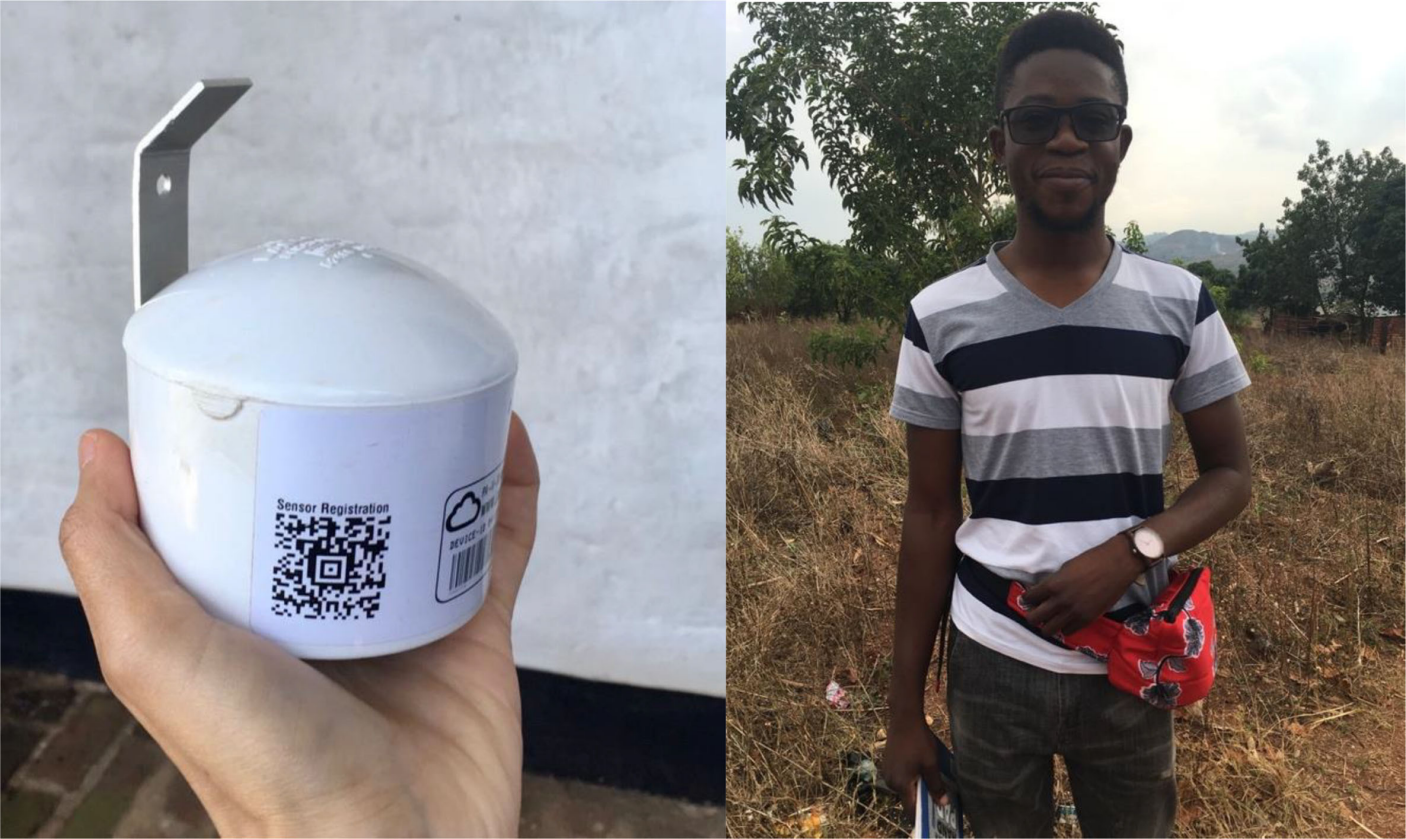
Largest monitor size and an example of a waist bag with monitors inside (worn by research assistant).

Participant observation formed the study foundation, with the researcher, accompanied by the research assistant and/or fieldworker, spending time in households and participating in cooking, farming and other daily activities. This household-based element allowed a graded introduction to the community. Each household observation lasted about 2–3 weeks, affording deep insights into the contexts in which smoke exists in the village. Mobile air quality monitors carried by researchers in small waist bags during this period (Figure 1) gave quantitative estimates of differential exposures to airborne particulate matter by time, place, person and activity.

To further develop these quantitative data, volunteers from a few participant observation households agreed to carry monitors overnight after researchers left the household. A short data review the following morning helped to identify exposure peaks and collect information about smoke sources. After identifying issues, largely around insufficient data, we amended the study protocol to allow further sampling in a separate, extended group of individuals (not previously involved in participant observation), each carrying the monitors for stand-alone 24-h periods. Inclusion of these additional study participants introduced further complexities around participant compensation.

### Participant compensation aspects

Initial plans were for a proportion of the research funds to be set aside as a ‘community compensation’ fund rather than individual monetary compensation, the nature of this fund being confirmed once more contextual information about the village emerged. We decided on this benefit-sharing approach in view of the inclusive nature of the project and recognising traditional African community-based value systems.^29^^–^^31^

The decision to provide compensation per se was informed by the fact that ‘aspirational benefits’ from the research (cleaner air and improved health) were not felt priorities for participants, at least at the project's start. Compensation thus allowed us to acknowledge and reciprocate burdens placed on community members in contributing to the project and to balance benefits to the research team.^31^^,^^32^

On application for in-country ethical approval, we learned that Malawian ethics committee guidance required monetary participant compensation amounting to US$10, although the distribution of this was not specified.^33^ In view of the varying forms of research involvement in different components (Table 1), specific decisions regarding compensation were therefore required. Together with senior Malawian researchers, we approached these decisions with the intention of maximising good (fair and appropriate compensation) and minimising harm. Plans around specific compensation payments were then written into the protocol, with these being taken from a sum of money set aside for compensation in the project budget. The remaining sum (following individual and household compensation payments) was used as community compensation at the end of the project.

For initial household-based participant observations, 8000 MK, equating to slightly more than US$10, was provided to each household in recognition of the potential disruptions caused by researchers’ prolonged presence. Compensation was on a household rather than individual basis, as activities were based around the household unit and because included members of any household often varied from day to day. Although this meant the 8000 MK sum was spread over a number of household members, this was deemed fair.

In addition, a set contribution was made to cover the food required to extend daily meals to the researchers present in the household. This sum (equating to 2,000 MK, approximately US$2.75 per day) was based on food prices and approximate portions per person, but with a margin to ensure (more than) adequate reimbursement at all times. These sums of money were presented with explanations of their differing reasons: the first as a way of acknowledging participants’ involvement and thanking them for their time and inconvenience and the second as direct reimbursement for money spent on the researchers’ food.

In the initial participant observation households, volunteers who continued to carry monitors did not receive extra monetary compensation. At this point there were no plans for additional personal air quality monitoring, so the above constituted the entirety of the proposed individual/household payments. Developments in the protocol with repercussions for compensation are now described.

## Results

### Intermediate study outcomes

Household observations and concomitant air quality monitoring progressed smoothly with development of good research relationships and widespread positive responses. Compensation was gratefully received and many people actively volunteered their households for involvement—more than was possible to include in the time frame. The extent to which this enthusiasm to participate was motivated by compensation payments remains uncertain.

Although intended research beneficiaries were members of the village themselves, aspirational research benefits (cleaner air) did not drive involvement: as the ethnography found, smoke was not generally seen as a problem for residents, who had more pressing daily concerns. The inclination to help a stranger, however, was undoubtedly a motivator of participation for many. Other factors may have included the novelty of having a foreign researcher in the household assisting with chores or anticipation of unarticulated benefit stemming from association with a research team from a well-funded institution.

The research team's presence was on a number of occasions linked to ‘good things coming to the village’ (as expressed by the chief during an early village meeting). Such thoughts may be shaped by experiences or accounts of research involvement in the region, with contributions from comparatively rich research institutions benefitting people living in extreme economic vulnerability.^34^ While there had been no recent research in this study area, there was widespread awareness of the Cooking and Pneumonia Study (CAPS), a large trial of relatively expensive solar-powered cookstoves in the nearby Chikwawa region.^35^ These stoves were often cited as examples of ‘clean cooking’ during consultation discussions. Linked with our research institution, CAPS and its ancillary studies afforded compensation and benefits to participants, including cookstoves, pots and monetary compensation for various forms of involvement.^36^

On proposing the extended plan of including additional participants to carry air quality monitors, feedback from community members via the resident fieldworker indicated discomfort around the expectation that these individuals would participate without receiving any financial compensation. Specifically, some felt it would be unjust to expect this when other participants had received money for their contributions. As researchers, we acknowledged this concern and the proposal was amended to incorporate compensation of 8000 MK for each person involved in stand-alone air quality monitoring.

This was well received by the community and a large number of individuals then volunteered to carry monitors, although a few still declined. Outright refusal to participate was rare throughout the study, and in the few cases where reasons were given, these were primarily not having enough time. Our ethnographic observations in general revealed a widespread willingness to help and reluctance to appear obstructive, somewhat obscuring findings on motivators and deterrents to participation. Unease around the monitoring equipment was occasionally seen however, e.g. when one couple refused to touch the monitors on their demonstration.

### Participant compensation decision-making

In our decisions around individual compensation, we aimed to maximise benefit and minimise harm. This involved a number of considerations, outlined here as a basis for the wider ethical debate.

We compensated participants for disruption of their daily activities, engagement with outsiders and discomfort or inconvenience of carrying a monitor, among other factors, as well as to demonstrate our appreciation for their involvement.

Our presence in households as participant-observers often slowed down chores such as food preparation and necessitated explanations to passers-by (at the local market for instance). Furthermore, despite our appeals to be treated as ordinary household members, extra efforts were frequently made to welcome us, most visibly in terms of the frequency and substance of meals prepared when we were present. While money spent on researchers’ food was reimbursed, the extra compensation recognised these additional burdens that our presence entailed.

Monitors, although quite small, may have been troublesome to carry, particularly in the context of daily physical work, and carriers took care of these instruments entrusted to them. They may also have attracted unwanted attention, particularly for earlier volunteers at a time when residents were perhaps less familiar with the devices. To an extent, monitors also constituted a breach of confidentiality for the wearers (although in practice participants were very open about study involvement). A degree of compensation for such disruptions could again represent one way of recognising these burdens and showing respect to participants.

Community members themselves raised the issue of comparative justice, suggesting that economic compensation should be provided for carrying the monitors. This demonstrated how a communities’ judgements of inconvenience or disruption and perspectives regarding fairness of compensation can inform such decisions.

We also attempted in our decisions to consider ‘fairness across research settings’: how participants contributing in similar ways in similar studies in different geographical locations might be compensated differently, although complexities relating to our use of mixed research methods, including participant observation, made this comparison difficult. Ethnographic studies vary widely in their approaches, with ‘appropriate’ compensation representing a culturally situated concept, an ongoing negotiated process involving the researcher and population.^37^

Potential risks associated with monetary compensation also featured in our deliberations. Our ethnography revealed how lives in the village are shaped by a resilience to profound economic poverty, which influences daily priorities and perspectives. In this context, US$10 is a comparatively large amount of money, carrying the potential to cause disruption within and between households and, as some have proposed, ‘undue inducement’ to research participation.^10^^,^^11^^,^^14^ In our study, deep and honest engagement with residents and regular discussions with the resident fieldworker afforded an extra level of community feedback, and we saw no evidence of disruption or undue inducement in the study, although the possibility of undetected low-level disruption within the community remains.

Finally, we considered the risk of comparatively large amounts of monetary compensation altering local expectations of compensation, thus negatively impacting local researchers or studies with more limited funding. Our decision regarding the compensation amount (in terms of the core sum provided) was set by the institutional research board, allowing a degree of consistency across all health-related studies in the area. In making supplementary decisions around the distribution of this sum (for instance, in terms of the decision to allow 8000 MK for each household in the first component but 8000 MK per participant in the air quality monitoring component) we were guided by community views, allowing some flexibility and contextual responsiveness.

## Discussion

We now analyse the key ethical issues surrounding participant compensation in the current study in the context of four values in transnational research proposed by Schroeder et al.^7^: fairness, care, honesty and respect. These values and relevant aspects of the study to each are outlined in Table 2, with more in-depth explorations below.

**Table 2.** Four values in transnational research and aspects of the current study relevant to each

Value	Relevant aspects
Fairness	Relative benefits to researchers and participants
	Amount of compensation in relation to participant burden
	Comparative justice between participants involved in different components of the study
	Comparative justice between participants in similar studies in different locations
Care	Prioritising participants’ welfare: potential for ‘undue influence on participation’ or community/household disruption caused by monetary compensation
	Role of community perspectives in decisions around participant compensation
	Awareness of the potential effects of historical and political contexts and power differentials with those in positions of power safeguarding the interests of relatively disempowered participants
Honesty	Clarity and honesty of consent processes
	Awareness of ongoing, renegotiated nature of consent
	Community engagement throughout project introduction and implementation
Respect	Respect for existing social structures in the setting
	Initial engagement of chief and key village stakeholders in allowing project to go ahead
	Awareness of how cultural norms and values may influence research participation

Fairness, or justice, is considered an important value, but its interpretation is deeply contextual and implementation in transnational research practice can be complex and multidimensional.^38^^–^^40^ Wider discussions around risks of exploitation in transnational research address comparative consideration of the relative benefits for researchers and participants and, for research involving economic compensation, contemplation on appropriate levels of participant benefits.^9^^,^^38^

The question of ‘fair compensation’ is particularly important in our study. Incurred costs can easily be reimbursed, as in the case of contributions to cover participants’ money spent on researchers’ food during household participant observations. More abstract burdens, such as the researchers’ presence for long periods or carrying of monitors, are more complex to value. Approaches to these decisions include market-driven as well as more distributive perspectives, the latter of which aim to manage underlying structural inequities in transnational research.^40^^–^^42^

In reference to our research experience in implementing a community-wide project, questions of which participants to compensate and how introduced additional levels of complexity. Our village-level compensation proposal acknowledged the typical community-centred value systems existing in African settings such as Malawi^29^^–^^31^ and was in keeping with evidence suggesting its use for collateral non-monetary benefits such as healthcare support.^14^^,^^43^

Through responsiveness to community voices we learned that village-level compensation could not replace individual compensation for certain participation types, a finding that is echoed in the literature from similar settings.^43^ This brought in an additional aspect of fairness, that of justice across participants involved in different study components.

In attempting to compare research contributions and make these decisions we found that our perceptions of fairness did not always match those of community members. Our assumption that carrying personal air quality monitors would be relatively simple—not requiring monetary compensation—was not supported by local residents’ views. This was perhaps understandable in view of the above-mentioned juxtaposition between how research benefits researchers and participants. In a project offering sufficient direct benefits to participants (from the research intervention), or not involving the same degrees of inequity (e.g. with participants more heavily invested in the research aim itself and/or with fewer competing priorities), this balance of interests might be different.

In the current situation, however, where power imbalances left decision makers (usually senior researchers) able to make judgements, leaving a relatively disempowered population to respond by agreeing to participate or not, ethics dumping was a real risk. Our study design allowed us to solicit community views and alter protocols accordingly, but other studies might require different approaches to ascertaining locally appropriate practices and integrating them into research plans.^13^^,^^27^

In setting compensation levels, we were bound by current/local institutional guidance. While accounting to some extent for differences in research procedure invasiveness, these remain relatively blunt tools with no allowance for potential ‘social invasiveness’ of, for instance, prolonged immersive participant observation.^33^ Such rates allow equal compensation across all similar research participants nationally but fail to fully account for certain, particularly qualitative, research methods and circumstances and can potentially disadvantage lesser-funded researchers.^14^^,^^33^^,^^44^

An alternative approach for determining fair compensation is for investigators to seek case-by-case advice from local research ethics committees applying contextual knowledge.^27^ One of our advisors cites experiences of using locally based non-governmental organisations to support and inform compensation decisions, again in an independent capacity (D. Schroeder, written communication, April 2020). Lastly, community representatives and patient groups could contribute to decisions independent of ethics review boards or research institutions.^13^^,^^45^^,^^46^ In view of the great variation in research study types and approaches, these decisions must be made by individual research teams.

The provision of economic compensation, particularly in a context of poverty, has been associated with risks of unduly influencing potential participants and of conflicts within households and communities.^10^^,^^11^^,^^14^ The concept of ethics dumping applies again here, where lower standards of ethical scrutiny in low-income settings could leave populations open to the adverse consequences of poor compensation practices. Such risks can be considered under the category of care, where, it is said, the responsibility of researchers is to ‘take care of the interests of those enrolled in research studies to the extent that one always prioritises their welfare over any other goals’.^7^

The Pamodzi research assistant provided insights into aspects of Malawian culture potentially amplifying risk of participant coercion, such as the widespread norm against actively opposing an offer (e.g. refusing research participation). Existing power differentials and colonial histories further this risk. The first step in ensuring ‘care’ was to recognise these inequalities and put provisions in place to mitigate these possibilities.

Initial discussions with key community leaders provided an extra safeguard for potentially vulnerable residents.^27^^,^^47^^,^^48^ While ‘community approval’ can itself be coercive in authoritarian settings and without adequate contextual understanding, we ensured that these initial approvals never replaced or compromised empowered individual consent processes and ongoing equitable engagement with communities: again vital in protecting research participants’ interests.^49^

This relates to the third value: honesty. Where honesty and care combine, researchers recognise the true nature of consent as a continuous renegotiated process throughout the research period.^50^^,^^51^ This open communication and community responsiveness—with a central role for the local fieldworker—is vital to ensuring continuing fair research practices in participation and compensation.

While our project entailed specific engagement mechanisms, larger research endeavours in similar settings have used community advisory groups to facilitate feedback.^46^^,^^52^^–^^54^ Complexities in terms of roles and relationships must be navigated here to ensure effective representation for potentially vulnerable participants and empowerment of community representatives to be advocates rather than enablers of research implementation.^45^^,^^55^

The final value to consider, that of respect, must particularly underlie the entirety of a research process from planning through implementation and beyond, and demands deep contextual engagement. Our project would not have been successful without Malawian team members at all levels, with the resident fieldworker—a lifelong member of the village with extended family also living in the village—being particularly valuable in promoting participant perspectives. Respectful research conduct also meant acknowledging existing local social structures, including traditional leadership, religious leaders and local health volunteers.^56^ Our project particularly recognised and respected the social position of the village chief, while not letting it override individual decision-making, a contrast to the exploitation of the chief's power in the accounts of colonial rule.^21^^,^^22^

Lastly, our approaches to participation and consent recognised potential differences in ethical approaches between typically individualised perspectives of many in the Global North and classical African collective bioethics.^57^ This strengthened our commitment to facilitating collective discussion and decision-making. Avoidance of this aspect of ethics dumping (failure to consider the sociocultural values of the research context) may again look different across research projects and requiring a ‘respect’ for the research setting and participants’ values throughout.

Our article used a case study to explore wider ethical issues, discussing ways to improve responsive and responsible research practice. While we aimed to identify alternative examples of ethical implementation of the values where our own were lacking or potentially challenging to reproduce, this was not always possible. Ultimately researchers bear the burden for determining ethically appropriate practice in individual research settings and study types.

While the focus of discussion has been transnational research, with the attendant contexts of post-colonialism and inequity, some themes will also be applicable to research elsewhere. The biomedical establishment has traditionally assumed a sense of ‘expert knowledge’ from which lay participants are excluded. This then speaks to a power imbalance between research and participant, even in the UK, which makes relevant much of the earlier discussion around equitable research conduct. For participants in circumstances of relative economic deprivation, this inequality becomes more marked, increasing potential vulnerability to research exploitation.

Based on the values examined above, the TRUST project, a multinational collaborative initiative aimed at improving adherence to high ethical standards globally and countering ethics dumping, proposed the first comprehensive global code of conduct to guide researchers in transnational research settings. Accompanied by supporting tools and materials, the code makes individuals and communities in the Global South aware of what they should expect in terms of fairness, care, honesty and respect and assists researchers in contemplating such ethical issues.^58^^,^^59^ We found the values set forth in this code very helpful, particularly in terms of organising our thoughts on ethical research approaches and considering how such approaches might be applied in future studies. We suggest that these values could assist others who aim for equitable research partnerships.

## Conclusions

Issues of participant compensation in transnational health research are often negotiated within contexts of economic inequity and complex power dynamics. Colonial histories and their enduring influences also shape these research environments. Risks of participant exploitation in these contexts must be taken seriously, although perspectives differ on how best to manage these risks.

The case above illustrates how the values of fairness, care, honesty and respect can be used to understand and respond to specific issues relating to participant compensation. Key recommendations concern the importance of meaningful engagement with study populations, with community insights contributing to study planning and implementation throughout the research process.

## Data Availability

The current paper is based on authors' experiences in conducting an ethnographic study. The ethnographic datasets on which this paper is based are not publicly available due to the levels of detail involved, making descriptions of participants potentially identifiable even after the removal of direct personal identifiers, and the inclusion of potentially sensitive participant data. The ethnographic data are however available from the corresponding author on reasonable request.
